# Screen time, physical activity, sleep, and depression risk in adolescents: an observational study based on the compositional isotemporal substitution model

**DOI:** 10.3389/fpubh.2026.1782512

**Published:** 2026-05-04

**Authors:** Hao Guo, Mengting Man, Zheng Hu, Yun Liu, Wen Chen, Sainan Wang, Xinmiao Tang, Anyi Geng, Wenhua Ruan, Wenzhuo Xu, Kele Jiang, Haiyan Shi, Jun Du, Guifang Jin, Xiaopeng Zhang, Zhihua Zhang

**Affiliations:** 1 Department of Epidemiology and Biostatistics, School of Public Health, Anhui Medical University, Hefei, Anhui, China; 2 Hefei 168 High School, Hefei, Anhui, China; 3 The People's Hospital of Chizhou, Chizhou, Anhui, China; 4 Hefei Center for Disease Control and Prevention, Hefei, Anhui, China; 5 Department of Public Health and Health Administration, Clinical College of Anhui Medical University, Hefei, Anhui, China

**Keywords:** adolescents, compositional isotemporal substitution model, depression, physical activity, screen time

## Abstract

**Background:**

Adolescent depression is linked to daily activities like screen use, physical activity, and sleep. Most studies examine these factors separately. This study investigates the relationship between screen time, physical activity, sleep, and depressive symptoms in adolescents using compositional isotemporal substitution models (CISM).

**Methods:**

A cross-sectional study collected data from 6,666 adolescents in Hefei, China, using self-administered questionnaires. Compositional Data Analysis was used to examine 24-h activity patterns and their association with depression. A compositional linear regression model was constructed to explore the relationships between screen time, low-intensity (LPA) and moderate-to-vigorous physical activity (MVPA), sleep (SLP), and adolescent depression. The CISM was then applied to analyze the effects of reallocating and substituting time between different activities on depression scores.

**Results:**

The compositional linear regression model revealed a significant positive correlation between screen time and depression relative to the remaining activities (β_ST_ = 0.693, *P* < 0.001), while LPA, MVPA and SLP showed significant negative correlations with depression relative to the remaining activities (β_LPA_ = −0.132, *P* < 0.05, β_MVPA_ = −0.293, *P* < 0.001, β_SLP_ = −0.981, *P* < 0.001). Using a 10-min substitution as an example, replacing MVPA, LPA, or SLP with ST increased depression scores by 0.09, 0.06 and 0.03 points, respectively. Conversely, replacing ST with MVPA, LPA, or SLP decreased scores by 0.07, 0.05 and 0.03 points, respectively. Longer substitution durations amplified these effects.

**Conclusion:**

The study highlights that reducing screen time and increasing physical activity or sleep can help alleviate depressive symptoms in adolescents.

## 1 Introduction

Depressive disorder (also known as depression) is a common mental disorder characterized by persistent low mood and a loss of interest or pleasure in activities (1). Adolescence is a transitional period between childhood and adulthood, during which physiological, psychosocial, and cognitive changes make adolescents more susceptible to psychological problems (2), with depression being particularly common. Research has found that some cases of depression can be traced back to childhood and increase dramatically during adolescence (3). It is estimated that nearly 14% of adolescents meet the diagnostic criteria for depression before the age of 18 (4). By the age of 19, approximately 25% of adolescents have experienced at least one depressive episode (5). In the United States, suicide has become the second leading cause of death among adolescents aged 12 to 17. Epidemiological studies show that 43% to 90% of adolescent suicide victims had at least one mental disorder at the time of death, with depression being the most common (6). Furthermore, depression is not only a major cause of disability and impairment worldwide (7), but also a major contributor to the global disease burden (8).

Depression results from multiple factors, including genetic, biological, environmental, and behavioral influences. Adolescents' daily behaviors, such as physical activity (PA), sedentary behavior (SB), and sleep (SLP), are potential depression factors. SB, including screen use, leisure-time sitting, and work-related sitting (9), is especially notable. The rise in screen time (ST) is a key feature of modern society, making its impacts a crucial issue in adolescent health research.

Regarding the definition of ST, most scholars believe that it is a metric for measuring the time individuals spend using electronic screens. Tang et al. (10), in their study on the relationship between ST and mental health in young people, stated that “ST” is a general term encompassing various devices (such as computers, televisions, and mobile phones) and their uses (such as gaming and social communication). Based on previous research, this study defines ST as the total duration spent watching or using electronic devices (such as televisions, computers, mobile phones, tablets, video game consoles, e-readers, and other electronic devices).

Although new electronic devices may offer potential benefits, children and adolescents are spending more time on screen-based activities than ever before (11). The World Health Organization (WHO) recommends that children and young people's recreational screen time be limited to no more than 2 h per day, but the majority not adhere to this guideline (12). A large-scale longitudinal study in the United States on brain development and child well-being found that increased ST is closely associated with depression and anxiety (13). Furthermore, adolescents who engage in more than 4 h of passive ST per day are more likely to meet the diagnostic criteria for major depressive episodes, social phobia, and generalized anxiety disorder (14). In the course of an adolescent's daily activities, other behavioral patterns, such as PA and SLP, also have significant impacts on depression. These behavioral patterns are interconnected and mutually influential, collectively shaping the adolescent's mental health status.

In contrast to ST, PA offers a positive behavioral choice, and its relationship with depression has become a focal point of research. PA is typically defined as any type of bodily movement produced by skeletal muscle tissue. PA not only benefits physical health but also promotes mental health through various pathways. A meta-analysis of a prospective study reported that individuals with higher levels of PA were 17% less likely to develop depression compared to those with lower PA levels (15). Additionally, an 11-year follow-up study found that exercising for an hour a week can reduce the risk of depression by 12% (16).

When studying the association between adolescent behaviors and depression, SLP also plays a crucial role as an important health behavior pattern. Healthy sleep patterns help maintain good physical health, immune function, mental health, and academic performance (17), whereas poor sleep is a direct catalyst for the development of emotional difficulties and affective disorders (18). A meta-analysis revealed a close relationship between depressive symptoms and sleep quality in children and adolescents (19). Furthermore, reports suggest that adolescents with sleep disorders are at a higher risk of developing depression later in life (20).

Although numerous studies have revealed the individual effects of ST, PA and SLP on adolescent depression, these studies either focus solely on ST or PA, without considering the intrinsic relationships between these behaviors (21). Due to the fixed total of 24 h per day, a competitive and trade-off relationship exists between screen time, physical activity, and sleep. An increase in time devoted to one behavior will inevitably reduce time available for the others, and such interactions cannot be ignored (22). To address issues related to time reallocation and behavioral substitution, the compositional isotemporal substitution model (CISM) is adopted in this study. CISM addresses the constrained nature of 24-h time-use data by treating 24-h activity behaviors as compositional data and using Compositional Data Analysis (CoDA) methods, which allow for the transformation from simplex to Euclidean space, addressing the constrained nature of 24-h time use data. It the estimation of changes in health indicators after reallocating time between behaviors, rather than merely exploring the effect of changing a single activity on health outcomes (22).

To date, many studies have used CISM to explore the relationship between activity-behavior time reallocation and adolescent health outcomes. While CISM has been widely used to investigate the effects of time allocation between PA, SB, and SLP on health outcomes, existing studies often categorize screen-based behaviors simply as sedentary behaviors, overlooking their unique impact on health as a distinct behavioral pattern. The unique value of this study lies in separating screen time from the broader category of sedentary behavior and treating it as an independent behavioral component. Using the CISM model, this study can precisely quantify the specific impact on depressive symptoms when screen time is substituted with physical activity of different intensities and sleep. The increase in adolescent ST may not only directly affect depressive symptoms but may also indirectly alter health outcomes by compressing PA and SLP time. However, few studies have used CISM to separately explore the potential impact of reallocating ST, PA, and SLP on adolescent depression. This study adopts this perspective, aiming to use CISM to evaluate the impact of the mutual substitution of screen use, PA, and SLP on adolescent depression using CISM. It further seeks to reveal the unique role of ST and its potential pathways, providing scientific evidence for adolescent mental health interventions and behavioral pattern optimization, while also offering guidance for public health practices.

## 2 Materials and methods

### 2.1 Study participants

**Figure 1:**
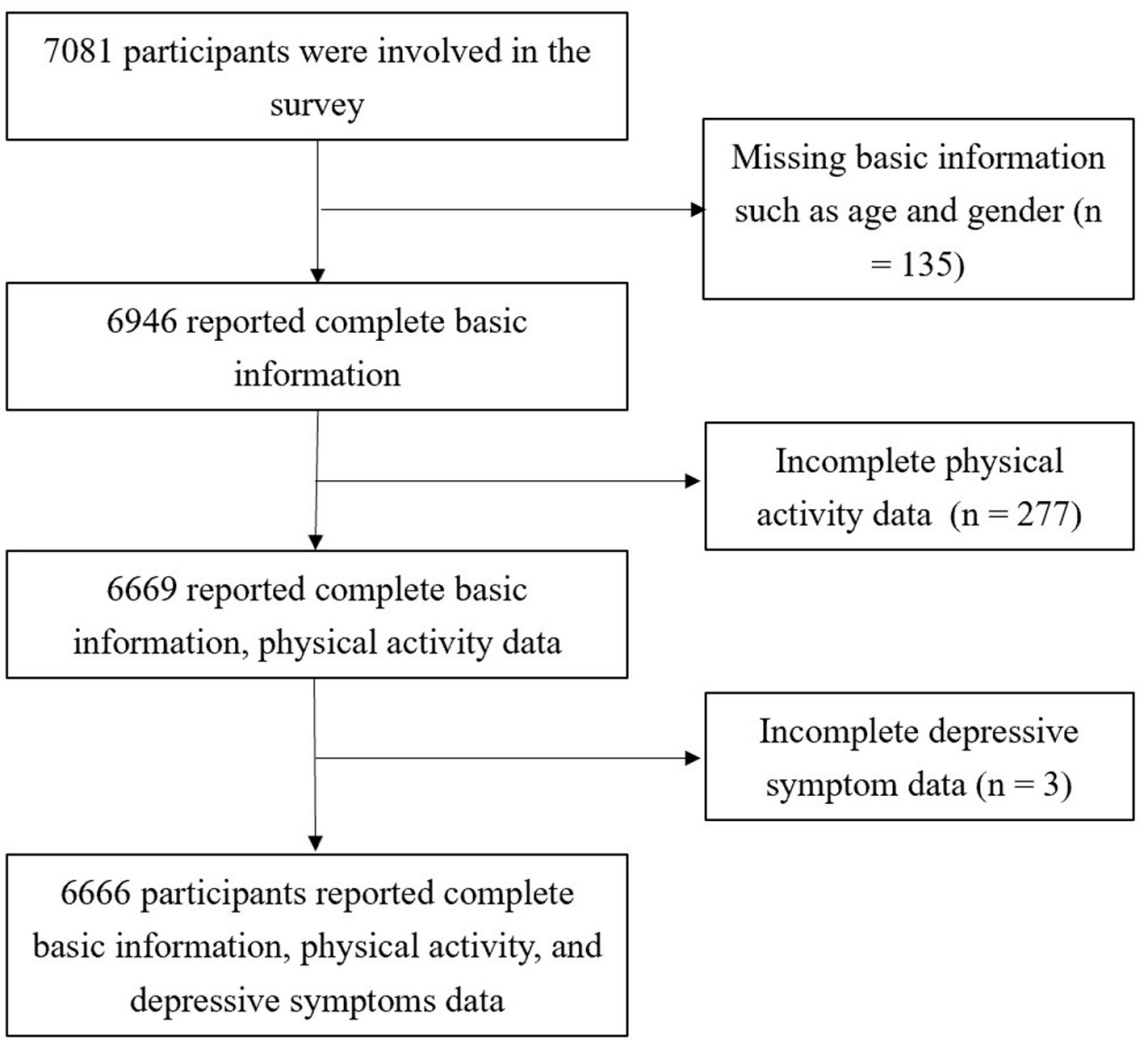
Research participant flowchart.

In this study, from April to June in 2024, four districts (Yaohai District, Luyang District, Shushan District, and Baohe District) and Chaohu City (a county-level city) under the jurisdiction of Hefei City were selected. Four schools were randomly selected from each district (two middle schools and two high schools), totaling 20 schools. Using a stratified cluster sampling method, four classes from each of the first and second grades were randomly selected from each school, with a total of 7,081 students included in the study. Due to missing or invalid data, the final number of valid questionnaires included in the analysis was 6,666, yielding an effective response rate of 94.14%, as shown in Figure 1.

### 2.2 Study content and methods

In this study, paper questionnaires were used for on-site investigation. The questionnaires were distributed by the class teachers or teachers by the uniformly trained investigators after obtaining the informed consent of the subjects, and the students filled in the questionnaires on the spot in the school classroom. In the process of filling out, students can raise their hands if they have any questions, and the investigators will answer them according to the uniform standards. After completion, the investigator checked the completeness of the questionnaire on the spot and promptly withdrew it. Quality control measures included: in the questionnaire design stage, the scale suitable for the study population was selected by referring to domestic and foreign literature, and epidemiological experts were invited to review, and the formal questionnaire was perfected after pre-investigation. During the survey implementation stage, all investigators received unified training, including the purpose of the survey, procedures, precautions and emergency handling. The standardized language was used to introduce the requirements to ensure information consistency. EpiData 3.1 was used to establish the database in the stage of data entry and verification. Two people independently entered the data and checked the consistency.

The questionnaire used in this study was independently designed, integrating scales with high reliability and validity that are widely used both domestically and internationally. The content of the questionnaire covered adolescents' general information, PA levels, SLP, screen use, and depression status.

#### 2.2.1 General information

The general survey covered information including their grade, gender, date of birth, height, weight, academic performance, family composition, whether they are an only child, whether they are a day student or boarder, parental education level, and household per capita annual income.

#### 2.2.2 Physical activity level

The physical activity levels of the participants were assessed using the Chinese version of the International Physical Activity Questionnaire Short Form (IPAQ-SF) (23). This questionnaire is a self-reported measure based on recall of activity levels over the past 7 days, and it has been comprehensively validated in 12 countries/regions (24). The questionnaire contains seven questions aimed at collecting the daily time (in minutes) and weekly frequency (in days) of walking, moderate-intensity, and vigorous-intensity activities, as well as daily sedentary time. Since the WHO and most epidemiological studies recommend combining MPA with VPA, as they have similar positive effects on mental health, and in terms of statistical stability and interpretation, this combination can reduce the number of variables to avoid multicollinearity issues and make the model simpler and the results more comparable, therefore in this study, MPA and VPA were combined into MVPA.

#### 2.2.3 Sleep time

Sleep status was measured using the Pittsburgh Sleep Quality Index (PSQI), which was proposed by Buysse et al. (25) in 1989 and is one of the most widely used scales for measuring sleep quality. The Chinese version of the scale, translated and validated by Liu et al. (26), was used in this study to assess individuals' sleep quality over the past month, with a primary focus on the dimension of sleep duration. We selected the dimension of sleep duration as the quantitative indicator for sleep (SLP) because it is highly consistent with the 24-h time-use analytical framework adopted in this study. With the compositional isotemporal substitution model (CISM) as the core analytical method, this study focuses on the time allocation and mutual substitution effects of different activities (MVPA, LPA, ST, NSST, SLP) within a 24-h period. All behavioral indicators included in the analysis are quantified in minutes. Therefore, extracting the “sleep duration” dimension from the PSQI ensures the unified dimensionality of all behavioral indicators, meets the data format requirements of compositional data analysis (CoDA), and enables the direct comparison of time spent on different activities and the analysis of their substitution effects.

#### 2.2.4 Screen time

The screen Time section of the questionnaire was adapted from the study on screen time by Tang et al. (27). The reliability and validity of this part of the questionnaire were tested by Tang using Cronbach's α coefficient, with an internal consistency α coefficient of 0.847, indicating high reliability. To collect information on participants' daily screen time over the past week (including time spent on entertainment, social interaction, education, and other purposes), we asked them to report their screen time on school days and weekends, involving the use of televisions, computers, mobile phones, tablets, video game consoles, e-readers, and other electronic devices. The calculation method was as follows: screen time on school days was multiplied by five, and screen time on weekends was multiplied by 2. The total was then summed and divided by seven to calculate the average daily screen time over the past week.

#### 2.2.5 Depression status

The depression status of the participants was assessed using the Patient Health Questionnaire-9 (PHQ-9). This scale consists of nine items, each rated on a 4-point scale, ranging from 0 to 3 (0 = Not at all; 1 = Several days; 2 = More than half of the days; 3 = Nearly every day). The total score ranges from 0 to 27, with higher scores indicating more severe depression. Total scores of 0–4, 5–9, 10–14, and 15–27 represent no depression, mild depression, moderate depression, and severe depression, respectively. In this study, the Cronbach's α coefficient for the PHQ-9 was 0.904.

### 2.3 Statistical analysis

In the statistical analysis part, descriptive statistical analysis was used in order of application. The continuous variables conforming to normal distribution were described by mean and standard deviation, the continuous variables not conforming to normal distribution were described by median and interquartile range, and the categorical variables were described by frequency and constituent ratio. Then non-parametric tests were used to compare the differences in depression scores, screen time and physical activity among different demographic characteristics. Secondly, the component data analysis method was used to calculate the geometric mean and variation matrix of 24-h activity behavior to describe the central tendency and discrete tendency. After that, the component data were transformed from simplex space to Euclidean space by the isometric log ratio transformation for the application of traditional statistical methods. Then, a component linear regression model was constructed to analyze the effects of each activity relative to other behaviors with ILR coordinates as the independent variable and depression score as the dependent variable.

Finally, this study used the compositional isotemporal substitution model (CoDA) to process compositional data and analyze the effects of behavioral components on depression scores. Compositional data refers to data where the sum of components is a fixed total (e.g., 24 h) and typically includes three types of activities: PA, SB, and SLP. In this study, PA was divided into moderate-to-vigorous physical activity (MVPA) and light physical activity (LPA), and SB was reallocated into ST and non-screen-based sedentary time (NSST), with a particular focus on screen-based behaviors. The specific operation was based on the geometric mean of each activity as the baseline, a series of new activity combinations were systematically created, and the reallocation of time among all pairs of activities was simulated, such as 5, 10, 15, and 20 min. The new combination was substituted into the component linear regression model by Isometric Log Ratio (ILR) transformation to obtain the new predicted value, and the predicted value of the pre-replacement combination was subtracted to obtain the mean change in depression scores by time reallocation.

Existing statistical methods are mostly applicable to unconstrained data, so the Isometric Log Ratio (ILR) transformation in CoDA was used to convert the data from the simplex space to the Euclidean space, making it easier to apply traditional statistical analysis methods. Using the sequential binary partitioning method, activity behaviors were converted into ILR coordinates, as follows:


$$\begin{array}{l} z_{1}=\sqrt{\frac{4}{5}}\ln\frac{t_{MVPA}}{\sqrt[{4}]{t_{LPA}*t_{NSST}*t_{SLP}*t_{ST}}}\\ z_{2}=\sqrt{\frac{3}{4}}\ln\frac{t_{LPA}}{\sqrt[{3}]{t_{NSST}*t_{SLP}*t_{ST}}}\\ z_{3}=\sqrt{\frac{2}{3}}\ln\frac{t_{NSST}}{\sqrt[{2}]{t_{SLP}*t_{ST}}}\\ z_{4}=\sqrt{\frac{1}{2}}\ln\frac{t_{SLP}}{t_{ST}} \end{array}$$

In the regression model, the transformed ILR coordinates were used as independent variables, with the depression score as the dependent variable. The compositional linear regression model is constructed as: *y* = β_0_+β_1_*z*_1_+β_2_*z*_2_+β_3_*z*_3_+β_4_*z*_4_+ε. In this equation, β_1_β_2_β_3_, and β_4_ are regression coefficients, and ε is the random error term. Finally, the CISM was used to investigate the effect of reallocating the time of five compositional variables on the outcome variable (PHQ-9 score). For example, to evaluate the effect of replacing 10 min of ST with MVPA, a new activity combination was created: (G_MVPA − 10_, G_LPA_, G_ST+10_, G_NSST_, G_SLP_). The ILR transformation was applied to these combinations, and the regression model was used to calculate the change in PHQ-9 scores after the substitution.

## 4 Results

### 4.1 Depression status of study participants

A total of 6,666 adolescents were included in this study, of whom 53.81% were boys and 46.19% were girls. Grades were evenly distributed, accounting for about 25% each from grade 1 to grade 2. In terms of body mass index, 53.44% of the participants were normal weight, 28.95% were underweight, 12.27% were overweight, and 5.34% were obese. 67.21% of the children were not the only child, and 81.23% did not live in school. Their academic scores were mainly middle and upper middle, totaling 61.78%. The father's education level was junior high school or below (35.49%), and the mother's education level was junior high school or below (44.19%).

The average depression score of the participants was 6.18 ± 5.79. The distribution of depression severity was as follows: no depression (3,134 participants, 47%), mild depression (2,109 participants, 31.6%), moderate depression (794 participants, 11.9%), and severe depression (629 participants, 9.4%), as shown in Table 1. In Table 2, non-parametric tests (Mann–Whitney *U*-test or Kruskal–Wallis *H*-test) were used to compare the differences in screen time among different demographic characteristics, and the statistic *Z* or *H* value and the corresponding P value were used to determine the significance of the differences between groups. The analysis results indicated that variables such as gender, grade, BMI, academic performance, and family background were significantly associated with depression scores (*P* < 0.001). Specifically, female participants had significantly higher depression scores than male participants. Higher grades and higher BMI levels were associated with increased depression scores, and there was a significant difference in depression scores between boarding and non-boarding students. Additionally, adolescents from families with lower incomes had higher depression scores, as shown in Table 2.

**Table 1:** Depression score rating levels.

Gender	No depression	Mild depression	Moderate depression	Severe depression
Male	1,931	1,045	354	257
Female	1,203	1,064	440	372
Total number of people	3,134	2,109	794	629

**Table 2:** Differences in depression scores across different demographic characteristics.

Characteristics	Depression score	*Z*/H value	*P*
Gender			
Male	4.00 (1.00, 8.00)	−13.588	< 0.001
Female	6.00 (3.00, 10.00)		
Grade			
Grade 7	3.00 (1.00, 7.00)	207.865	< 0.001
Grade 8	4.00 (1.00, 8.00)		
Grade 10	6.00 (3.00, 9.00)		
Grade 11	6.00 (3.00, 9.00)		
BMI category			
Underweight	4.00 (1.00, 8.00)	31.750	< 0.001
Normal	5.00 (2.00, 9.00)		
Overweight	5.00 (2.00, 9.00)		
Obese	5.00 (2.00, 9.00)		
Only child			
Yes	4.00 (1.00, 8.00)	4.240	< 0.001
No	5.00 (2.00, 9.00)		
Boarding school			
Yes	6.00 (3.00, 9.00)	−5.133	< 0.001
No	5.00 (2.00, 9.00)		
Academic performance			
Low	7.00 (3.00, 13.00)	154.312	< 0.001
Below average	6.00 (2.00, 10.00)		
Average	5.00 (2.00, 9.00)		
Above average	4.00 (1.00, 8.00)		
Excellent	4.00 (1.00, 7.00)		
Father's educational level			
Junior high school or below	5.00 (2.00, 9.00)	24.579	< 0.001
High school	5.00 (2.00, 9.00)		
Vocational/Technical school	5.00 (2.00, 9.00)		
Associate degree	5.00 (2.00, 9.00)		
line Bachelor's degree	4.50 (1.00, 8.00)		
Graduate degree or above	4.00 (1.00, 8.00)		
Mother's educational level			
Junior high school or below	5.00 (2.00, 9.00)	32.454	< 0.001
High school	5.00 (2.00, 9.00)		
Vocational/Technical school	6.00 (2.00, 9.00)		
Associate degree	5.00 (2.00, 9.00)		
Bachelor's degree	4.00 (1.00, 8.00)		
Graduate degree or above	3.00 (0.00, 9.00)		
Annual household per capita income			
< 10,000	6.00 (2.00, 10.00)	26.250	< 0.001
10,000–29,999	5.00 (2.00, 9.00)		
30,000–49,999	5.00 (2.00, 9.00)		
50,000–69,999	5.00 (1.00, 8.75)		
70,000–89,999	5.00 (2.00, 8.00)		
90,000–109,999	4.00 (1.00, 8.00)		
≥110,000	5.00 (1.00, 8.00)		

### 4.2 Screen time

The analysis of compositional data in this study revealed significant differences in the geometric mean (in minutes) of screen time (ST) across different demographic groups, as shown in Table 3. Gender analysis indicated that females had significantly higher ST than males (*P* < 0.001). Significant statistical differences in ST were also observed across grades, BMI levels, and boarding status (*P* < 0.001). Specifically, ST increased with higher grades and BMI levels, while boarding students had relatively lower ST (*P* < 0.001). Moreover, variables such as academic performance, parental education level, and family income per capita were significantly correlated with ST (*P* < 0.001).

**Table 3:** Screen time across different demographic characteristics.

General situation	ST (G)	*Z*/H value	*P*
Gender			
Male	313.47	6.686	< 0.001
Female	328.12		
Grade			
Grade 7	270.72	347.128	< 0.001
Grade 8	280.99		
Grade 10	355.83		
Grade 11	383.14		
BMI category			
Underweight	299.36	26.186	< 0.001
Normal	321.60		
Overweight	348.22		
Obese	367.58		
Only child			
Yes	316.92	0.353	0.724
No	322.38		
Boarding school			
Yes	316.92	−4.873	< 0.001
No	322.38		
Academic performance			
Low	345.59	23.440	< 0.001
Below average	329.80		
Average	328.51		
Above average	306.42		
Excellent	308.16		
Father's educational level			
Junior high school or below	323.54	20.244	< 0.001
High school	338.40		
Vocational/Technical school	354.97		
Associate degree	318.83		
Bachelor's degree	283.36		
Graduate degree or above	292.41		
Mother's educational level			
Junior high school or below	332.61	44.846	< 0.001
High school	319.05		
Vocational/Technical school	352.20		
Associate degree	324.842		
Bachelor's degree	272.26		
Graduate degree or above	266.04		
Annual household per capita income			
< 10,000	312.39	27.706	< 0.001
10,000–29,999	345.97		
30,000–49,999	329.25		
50,000–69,999	301.51		
70,000–89,999	308.98		
90,000–109,999	337.79		
≥110,000	312.39		

ST, screen time.

**Figure 2:**
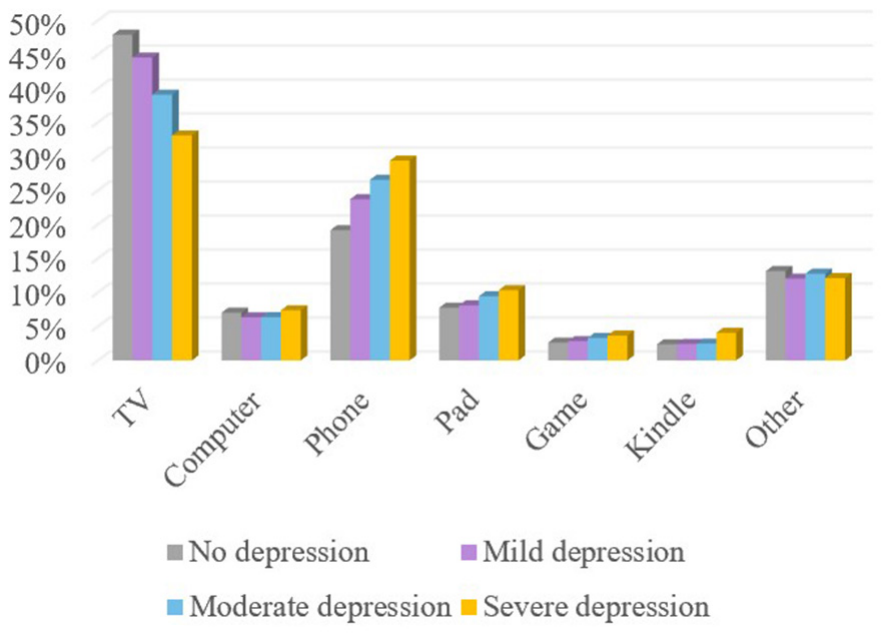
Proportion of adolescents using screen devices.

The usage of different electronic devices by adolescents is shown in Figure 2. Regardless of the severity of depression, the proportion of time spent on TV was consistently the highest, followed by mobile phone usage. As depression severity increased, the proportion of time spent using mobile phones, tablets, video game consoles, and e-readers all showed an upward trend.

### 4.3 Time distribution of adolescents' 24-h activity behaviors

#### 4.3.1 Central tendency

The 24-h activity behavior time for adolescents is composed of MVPA, LPA, ST, NSST, and SLP. The central tendency was described using geometric means, as shown in Table 4, which presents the geometric and arithmetic means along with their percentage of each activity time. Whether using geometric or arithmetic means, the percentage distribution of 24-h activity time is as follows: SLP > NSST > ST > MVPA > LPA.

**Table 4:** Geometric means of various physical activities in adolescents over 24 h.

Type of activity	Geometric mean (Min)	Percentage (%)	Arithmetic mean (Min)	Percentage (%)
MVPA	44.11	3.06	72.40	5.03
LPA	32.02	2.22	53.09	3.69
ST	320.59	22.26	380.10	26.40
NSST	385.04	26.74	395.02	27.43
SLP	658.24	45.72	539.39	37.45

MVPA, moderate-to-vigorous physical activity; LPA, low-intensity physical activity; ST, screen time; NSST, non-sedentary screen time; SLP, sleep.

#### 4.3.2 Dispersion tendency

The dispersion tendency of the 24-h activity behavior proportions is described using a variance-covariance matrix. Through the variation matrix, we quantified the relative differences between different activity behaviors. As shown in Table 5, the isometric log-ratio (ILR) variance between SLP and NSST is the smallest, at 0.780. In contrast, the variance between ST and MVPA is the largest, at 4.411, with MVPA showing the highest ILR variance compared to the other four activities.

**Table 5:** Pairwise log-ratio variance-covariance matrix of various physical activities in adolescents over 24 h.

Type of activity	MVPA	LPA	ST	NSST	SLP
MVPA	0	3.296999	4.411397	3.46981	2.618966
LPA	3.296999	0	3.355167	2.546195	1.797028
ST	4.411397	3.355167	0	2.963138	1.580719
NSST	3.46981	2.546195	2.963138	0	0.779976
SLP	2.618966	1.797028	1.580719	0.779976	0

MVPA, moderate-to-vigorous physical activity; LPA, low-intensity physical activity; ST, screen time; NSST, non-sedentary screen time; SLP, sleep.

### 4.4 Compositional linear regression of 24-h activity behaviors and depression scores

After controlling for variables included grade, sex, BMI class, performance rank, father's education level, mother's education level, and per capita household income, compositional linear regression analysis was conducted with each of the five activity components (MVPA, LPA, ST, NSST, and SLP) as independent variables and depression score as the dependent variable. The results of the regression analysis, shown in Table 6, reveal significant statistical associations between each activity component and depression scores. Specifically, MVPA, LPA, and SLP are negatively correlated with depression scores (β_MVPA_ = −0.293, *P* < 0.001; β_LPA_ = −0.132, *P* < 0.05; β_SLP_ = −0.981, *P* < 0.001), while ST and NSST are positively correlated with depression scores (β_ST_ = 0.693, *P* < 0.001; β_NSST_ = 0.712, *P* < 0.001).

**Table 6:** Compositional linear regression of 24-h activity components and depression scores in adolescents.

24-h activity behavior component	Regression coefficient (standard error)	*P*	Model *P*-value
MVPA vs. other components	−0.293 (0.051)	< 0.001	*P* < 0.001
LPA vs. other components	−0.132 (0.059)	< 0.05	
ST vs. other components	0.693 (0.064)	< 0.001	
NSST vs. other components	0.712 (0.091)	< 0.001	
SLP vs. other components	−0.981 (0.142)	< 0.001	

MVPA, moderate-to-vigorous physical activity; LPA, low-intensity physical activity; ST, screen time; NSST, non-sedentary screen time; SLP, sleep.

### 4.5 Impact of replacing different types of activities on depression scores

This study used the CISM to examine the impact of substituting ST with other activity behaviors on depression scores. When the substitution time was set to 10 min, the results showed that after adjusting for variables such as grade and gender, the substitution of ST for MVPA, LPA, and SLP had a statistically significant impact on depression scores. Specifically, substituting ST for MVPA, LPA, and SLP led to an increase in depression scores by 0.09 (95% CI = 0.06–0.11), 0.06 (95% CI = 0.02–0.10), and 0.03 (95% CI = 0.03–0.04) units, respectively, while substituting ST with NSST showed no statistically significant result. In contrast, replacing 10 min of MVPA, LPA, or SLP with ST led to a decrease in depression scores by 0.07 (95% CI = −0.09 to −0.05), 0.05 (95% CI = −0.08 to −0.02), and 0.03 (95% CI = −0.04 to −0.03) units, respectively, with NSST replacing ST also showing no statistical significance. As the substitution time increased, the impact of activity behavior substitution on depression scores became stronger. Table 7 shows in detail the results of the analysis of the effect of substituting different activity behaviors for each other on depression scores.

**Table 7:** The potential impact of substituting different activity behaviors on depression scores (95%CI).

Type of activity	MVPA ↓	LPA ↓	ST ↓	NSST ↓	SLP ↓
10 min					
MVPA **↑**		−0.01 (−0.06 to 0.04)	−0.07 (−0.09 to −0.05)^***^	−0.07 (−0.09 to −0.05)^***^	−0.04 (−0.06 to −0.02)^***^
LPA **↑**	0.04 (−0.01 to 0.08)		−0.05 (−0.08 to −0.02)^***^	−0.05 (−0.08 to −0.02)^***^	−0.02 (−0.05 to 0.01)
ST **↑**	0.09 (0.06 to 0.11)^***^	0.06 (0.02 to 0.10)^**^		0.00 (0.00 to 0.01)	0.03 (0.03 to 0.04)^***^
NSST **↑**	0.08 (0.06 to 0.11)^***^	0.06 (0.02 to 0.10)^**^	0.00 (−0.01 to 0.00)		0.03 (0.02 to 0.04)^***^
SLP **↑**	0.05 (0.03 to 0.08)^***^	0.03 (−0.01 to 0.07)	−0.03 (−0.04 to −0.03)^***^	−0.03 (−0.04 to −0.02)^***^	
20 min					
MVPA **↑**		0.02 (−0.10 to 0.13)	−0.14 (−0.17 to −0.10)^***^	−0.13 (−0.17 to −0.10)^***^	−0.07 (−0.11 to −0.03)^***^
LPA**↑**	0.10 (0.02 to 0.18)^*^		−0.10 (−0.15 to −0.05)^***^	−0.09 (−0.14 to −0.04)^***^	−0.03 (−0.08 to 0.02)
ST**↑**	0.20 (0.14 to 0.25)^***^	0.15 (0.05 to 0.25)^**^		0.00 (−0.01 to 0.01)	0.06 (0.05 to 0.08)^***^
NSST**↑**	0.19 (0.14 to 0.25)^***^	0.15 (0.05 to 0.25)^**^	−0.01 (−0.02 to 0.00)		0.06 (0.04 to 0.07)^***^
SLP**↑**	0.13 (0.08 to 0.19)^***^	0.09 (−0.01 to 0.19)	−0.07 (−0.08 to −0.05)^***^	−0.06 (−0.08 to −0.05)^***^	
30 min					
MVPA **↑**		0.19 (−0.11 to 0.49)	−0.20 (−0.24 to −0.15)^***^	−0.19 (−0.24 to −0.14)^***^	−0.10 (−0.15 to −0.04)^***^
LPA **↑**	0.22 (0.08 to 0.36)^**^		−0.14 (−0.21 to −0.07)^***^	−0.13 (−0.20 to −0.06)^***^	−0.04 (−0.11 to 0.04)
ST **↑**	0.35 (0.25 to 0.46)^***^	0.38 (0.10 to 0.67)^**^		0.00 (−0.01 to 0.02)	0.10 (0.08 to 0.12)^***^
NSST **↑**	0.35 (0.24 to 0.45)^***^	0.37 (0.09 to 0.66)^*^	−0.01 (−0.03 to 0.00)		0.09 (0.07 to 0.11)^***^
SLP **↑**	0.26 (0.15 to 0.36)^***^	0.29 (0.00 to 0.57)	−0.10 (−0.12 to −0.08)^***^	−0.09 (−0.11 to −0.07)^***^	

MVPA, moderate-to-vigorous physical activity; LPA, low-intensity physical activity; ST, screen time; NSST, non-sedentary screen time; SLP, sleep.

^*^P < 0.05;

^**^P < 0.01;

^***^P < 0.001.

**Figure 3:**
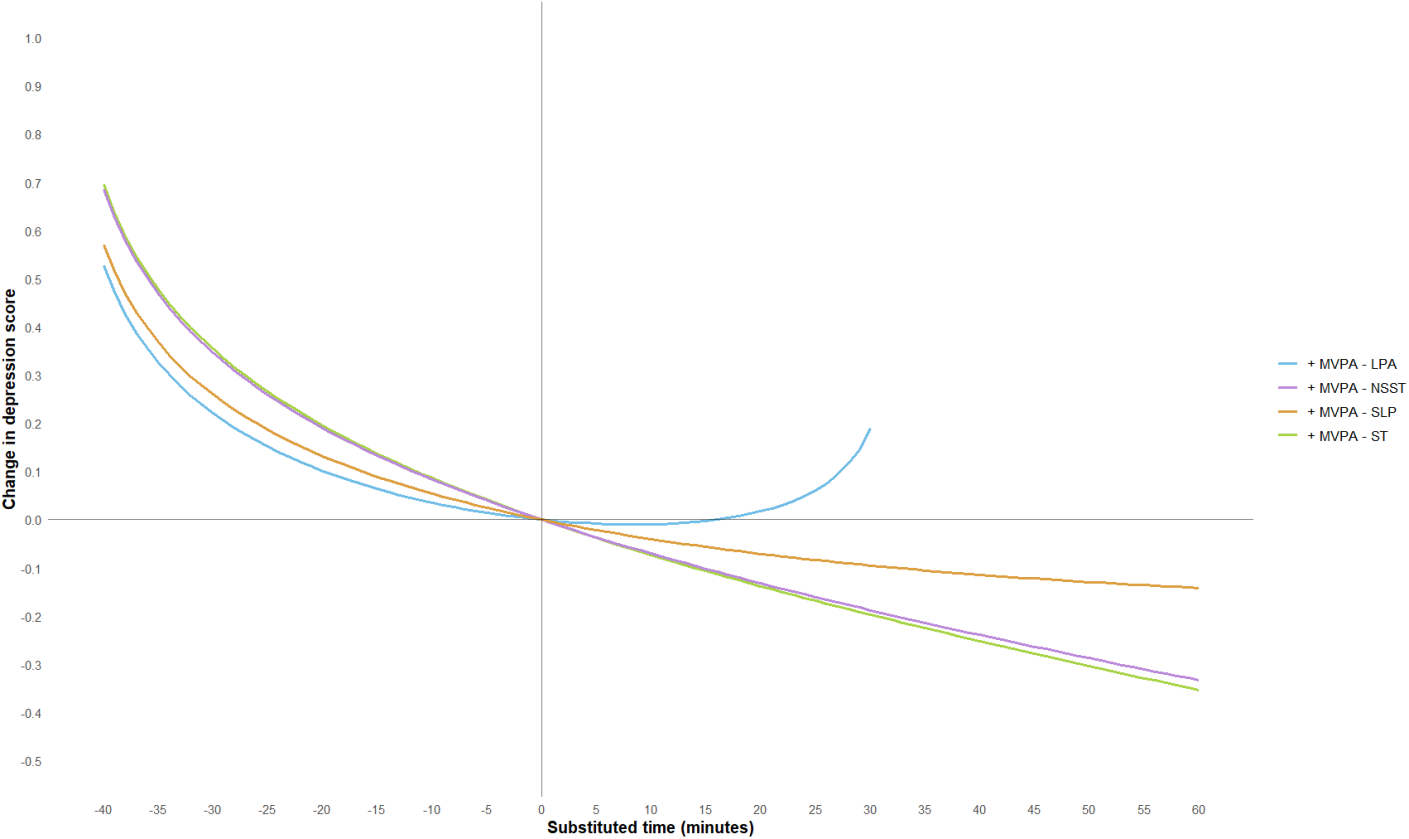
MVPA substitution impact on depression scores. MVPA, moderate-to-vigorous physical activity; LPA, low-intensity physical activity; NSST, non-sedentary screen time; SLP, sleep; ST, screen time.

**Figure 4:**
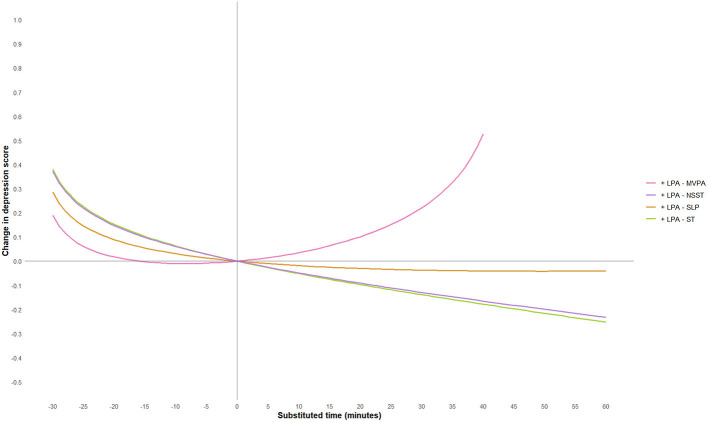
LPA substitution impact on depression scores. MVPA, moderate-to-vigorous physical activity; LPA, low-intensity physical activity; NSST, non-sedentary screen time; SLP, sleep; ST, screen time.

**Figure 5:**
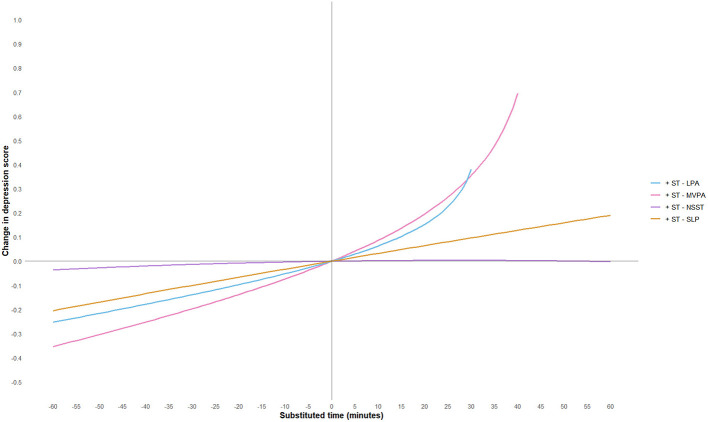
ST substitution impact on depression scores. MVPA, moderate-to-vigorous physical activity; LPA, low-intensity physical activity; NSST, non-sedentary screen time; SLP, sleep; ST, screen time.

**Figure 6:**
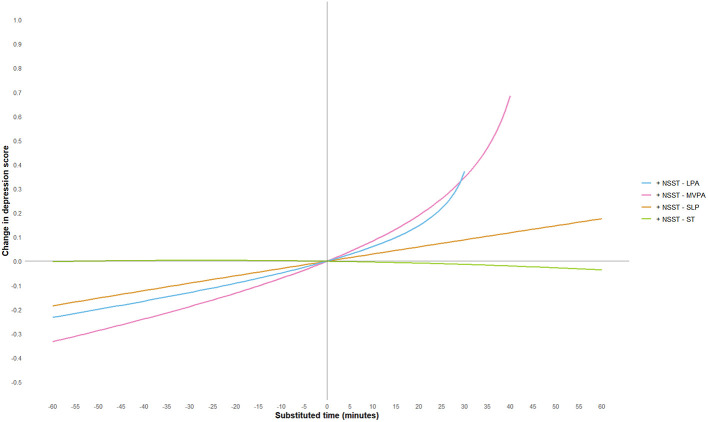
NSST substitution impact on depression scores. MVPA, moderate-to-vigorous physical activity; LPA, low-intensity physical activity; NSST, non-sedentary screen time; SLP, sleep, ST, screen time.

**Figure 7:**
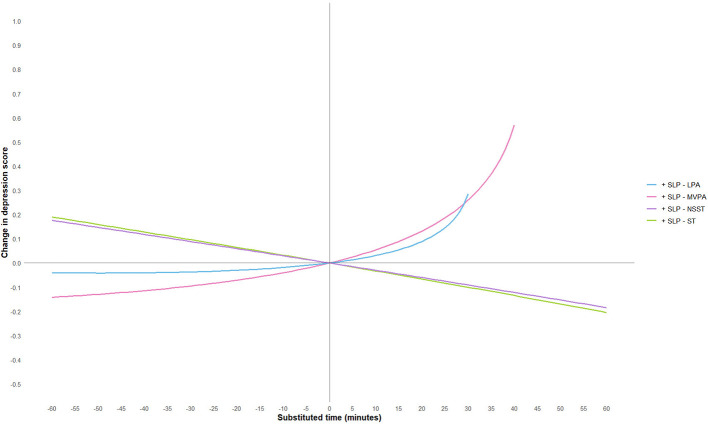
SLP substitution impact on depression scores. MVPA, moderate-to-vigorous physical activity; LPA, low-intensity physical activity; NSST, non-sedentary screen time; SLP, sleep; ST, screen time.

To further explore the effect of substituting different activity behaviors on depression scores, this study used a 5-min increment and plotted substitution curves for each activity with the other four behaviors within the range of −60 min to 60 min. However, since the geometric means of MVPA and LPA in this study were 44.11 and 32.02 min, respectively, and in accordance with the non-negative nature of compositional data, the substitution time ranges for these two activities were adjusted to −40 min to 60 min and −30 min to 60 min, respectively. The substitution results are shown in Figures 3–7.

## 5 Discussion

This study found that among 6,666 adolescents, the average depression score was 6.18, with 53% of participants experiencing some level of depressive symptoms. Using compositional data analysis, we observed that sleep occupied the largest proportion of adolescents' 24-h day (45.72%), followed by non-screen sedentary time (26.74%) and screen time (22.26%), while physical activity accounted for the smallest proportion (5.28%). The compositional linear regression model revealed that relative to other activities, screen time was significantly positively associated with depression (β = 0.693, P < 0.001), whereas light physical activity (β = −0.132, P < 0.05), moderate-to-vigorous physical activity (β = −0.293, P < 0.001), and sleep (β = −0.981, P < 0.001) were significantly negatively associated with depression. Furthermore, the compositional isotemporal substitution model demonstrated that reallocating just 10 min from screen time to moderate-to-vigorous physical activity could reduce depression scores by 0.07 units, while the opposite substitution increased scores by 0.09 units, with effects amplifying as substitution duration increased.

Adolescence is a critical period when the incidence of depression increases, which can have a profound impact on the growth, learning, and interpersonal relationships of children and adolescents (28, 29). In modern society, especially with the rapid development of information technology, the daily behavioral patterns of adolescents have changed, with sedentary and screen-based behaviors have become potential threats to both physical and mental health. With the widespread use of smart devices, adolescents' screen time has steadily increased, often replacing physical activity and sleep. High levels of physical activity, low levels of screen time, and appropriate sleep time are independently associated with fewer depressive symptoms in adolescents (30). These behaviors are interdependent, and therefore, the substitution effects between them need to be considered comprehensively. The CISM can estimate the substitution effects between different behaviors while controlling for confounding factors (31). This study aims to apply this model to explore the impact of time allocation between screen time, physical activity, and sleep on depressive symptoms, providing a theoretical basis for mental health interventions.

### 5.1 Adolescent depression status

This survey found that the average depression score of junior and senior high school students was 6.18, with 47.00% reporting no depression, 31.60% mild depression, 11.90% moderate depression, and 9.40% severe depression. This distribution is similar to findings from other regions (32–34). These studies suggest that mild depression is relatively common among adolescents, highlighting the need for early attention and intervention to prevent symptom worsening, which could affect academic performance, social relationships, and overall health. The study also found that girls had a higher rate of depression symptoms compared to boys, which is consistent with most studies (4, 35, 36). Biological differences, such as gene expression and neuroplasticity, may explain the gender disparities (37). Additionally, depression symptoms became more severe as grade levels increased, possibly due to rising academic pressure and psychological burden. The relationship between BMI and depression symptoms is more complex. Although adolescents with normal weight and those who are overweight or obese had higher depression scores, this contradicts some research results (38, 39), possibly due to societal expectations regarding appearance and adolescents' self-image anxiety. Family background factors, such as family structure, parental education level, and economic status, may also influence depression scores through their effects on the growing environment and psychological support.

### 5.2 Screen time among adolescents

The survey revealed significant differences in adolescents' ST across different demographic characteristics. First, girls spent more time on screens than boys, which may be related to differences in gender roles and interests. In terms of grade levels, senior high school students had longer screen time, possibly due to changes in academic pressure and entertainment needs. Adolescents with higher BMI, poorer academic performance, non-boarding status, and lower parental education levels generally had higher screen time, which aligns with Byun et al.'s study (40). Byun suggested that increased screen time may lead to prolonged sedentary time, thereby reducing calorie expenditure and affecting metabolism. Additionally, adolescents with poorer academic performance may use screen entertainment to relax, while boarding students, due to stricter time management, tend to have less screen time. Adolescents whose parents have lower education levels tend to spend more time on screens due to less supervision and limited educational resources.

Regarding the use of different screen devices, television always accounted for the highest proportion of screen time, followed by mobile phones, regardless of the severity of depression. The popularity of television and its entertainment functions, as well as the use of multimedia equipment in classrooms, may contribute to increased exposure to television screens among adolescents. Mobile phones, due to their convenience and social functions, serve as important tools for escaping reality and seeking emotional support, especially among adolescents with more severe depression.

### 5.3 Time allocation of adolescents' 24-h activity behaviors

This study explored the impact of adolescents' 24-h activity behavior time distribution on depressive symptoms, specifically focusing on MVPA, LPA, ST, NSST, and SLP. The analysis showed that SLP occupied the largest proportion of adolescents' daily activity time, followed by ST and NSST, while PA (including MVPA and LPA) took up less time, which is consistent with findings from most scholars (41–43). However, the specific time allocation for PA in this study differed from previous research. This study found that adolescents spent more time on MVPA than on LPA, whereas most past studies reported that LPA typically took more time (44, 45). This discrepancy may be related to measurement methods. This study used self-reported questionnaires that only recorded LPA (e.g., walking) lasting more than 10 min, which may have underestimated walking time. In contrast, other studies may have used more objective measurement tools, such as accelerometers or smart devices (31, 46) which can more accurately capture PA intensity and time allocation. Regarding ST, adolescents typically spent more than the WHO-recommended daily maximum of 2 h, and research in several countries has shown that adolescents' total screen time is generally excessive (47, 48). These phenomena highlight the need for more attention to interventions and management of adolescents' ST to promote their physical and mental health. Furthermore, analyzing the dispersion trend of adolescents' 24-h activity behaviors showed that MVPA had a larger isometric log-ratio variance, indicating that its time distribution has a more stable impact on health outcomes and is less influenced by other behaviors. This may be due to the specificity of MVPA, which is usually performed through dedicated exercise or sports activities (49), unlike screen behaviors or sleep, which are more easily influenced by daily life.

The pattern of time allocation observed in this study must be understood in the context of China's unique educational and cultural context. The considerable proportion of non-screen sedentary time (26.74%) may represent the large amount of time Chinese adolescents spend in classroom teaching, sitting to complete homework, and participating in evening self-study programs—a prominent feature of Chinese secondary education, where students typically stay in school until 9–10 p.m. The relatively low percentage of time spent on physical activity (5.28%) reflects the diminished importance of physical education in China's test-oriented education system, where physical education classes are often replaced by academic instruction, especially at the high school level. It must be considered that Chinese adolescents are increasingly using digital devices for academic purposes, including online courses and educational apps, thus blurring the distinction between recreational and educational use of screens. In addition, the phenomenon of “left-behind children” in families whose parents work in distant cities may lead to reduced parental supervision and thus excessive screen time. Together, these culture-specific factors explain why Chinese adolescents exhibit this particular pattern of time allocation.

In conclusion, this study found significant differences in adolescents' 24-h activity behavior time allocation, especially in terms of physical activity and screen time. To promote the physical and mental health of adolescents, efforts should be made to increase physical activity and effectively manage screen time.

### 5.4 Association between adolescents' 24-h activity behaviors and depression

The 24-h activity behavior patterns of adolescents not only reflect their daily habits but also have significant effects on mental health. This study used CoDA to explore the relationship between five main activity behaviors (MVPA, LPA, ST, NSST, and SLP) and depressive symptoms. Through compositional linear regression analysis, the results showed that, compared to other activity behaviors, LPA, MVPA, and SLP were significantly negatively correlated with depression scores, suggesting that these behaviors help alleviate depressive symptoms and have a protective effect. In contrast, ST and NSST were significantly positively correlated with depression scores, indicating that these behaviors may be risk factors for depressive symptoms. Previous studies have also shown that PA can activate the endogenous cannabinoid system, thereby reducing the risk of depression (50); insufficient sleep can disrupt emotional regulation, making it difficult for individuals to control negative emotions (51); and increased screen time can reduce physical activity, potentially increasing the risk of depression (52).

By comparing the regression coefficients, this study also found that the strength of the relationship between different activity behaviors and depression scores varied. Notably, the relationship between SLP and depression scores was the most significant among the five behaviors, suggesting that SLP duration may play an important role in regulating depression. Adequate sleep is crucial for adolescents' physical health, emotional stability, and academic performance (53). MVPA had a more significant effect on depression scores than LPA, which may be due to PA potentially influencing depressive symptoms through various socio-psychological and biological mechanisms, such as stimulating neural plasticity in brain areas associated with depression, reducing inflammation, or promoting self-esteem (54). NSST had a slightly stronger effect on depression than ST, possibly because non-screen sedentary behaviors encompass a wider range of lifestyle issues, such as prolonged inactivity, lack of exercise, and limited social interaction (55), which can lead to feelings of isolation and depression. In contrast, the diversity of screen use, such as electronic reading, may mitigate some of the negative impacts on mental health (56). More importantly, screen behaviors, represented by social media, can serve as channels for social connection among adolescents. Prior research has indicated that social media use helps fulfill individuals' needs for belonging and self-presentation (57, 58); by sustaining continuous connections with relatives, friends, and interest-based communities, it alleviates loneliness and enhances psychological adaptability. When individuals perceive stronger social support, their psychological resilience and stress-coping capacity improve accordingly, and this positive effect can counteract, to a certain extent, the adverse impacts of ST. Therefore, NSST exerts a more prominent impact on depression due to its greater lack of social interaction and cognitive benefits. In contrast, ST encompass positive functions such as social bonding and information acquisition, resulting in relatively weaker negative effects. Ultimately, this manifests as a slightly stronger association between NNST and depression compared to ST.

### 5.5 Relationship between time substitution of adolescents' activity behaviors and depressive symptoms

This study used the CISM to explore the interactions between different activity behaviors (MVPA, LPA, ST, NSST, SLP) in time substitution and their impact on depressive symptoms. Adolescent activity behaviors are not isolated but often exhibit substitution relationships, which traditional analytical methods fail to adequately assess. Therefore, we applied CISM to more comprehensively reveal the effects of substitution between different activities on depressive symptoms (59).

The study found that when ST substituted for LPA, MVPA, or SLP, depression scores significantly increased. Notably, when ST replaced MVPA, the increase in depression scores was most pronounced, and the effect became more significant as the substitution time increased. For example, when ST replaced 10 min of MVPA, depression scores increased by 0.09 units, and when the substitution time reached 40 min, depression scores increased by 0.70 units. This suggests that excessive ST, especially when it replaces physically active behaviors that help regulate emotions, may exacerbate depressive symptoms. This may be because MVPA (e.g., aerobic exercise) exerts an antidepressant effect through multiple pathways: on the one hand, MVPA can more effectively promote the release of neurotransmitters such as endocannabinoids (50), dopamine (60) and 5-hydroxytryptamine (61), enhance the functions of the reward pathway and the emotional regulation center, thereby directly improving mood and alleviating anhedonia and low mood; on the other hand, MVPA can reduce the levels of peripheral inflammatory factors, mitigate neuroinflammatory responses, and improve hippocampal neuroplasticity—these pathways are precisely the core pathological mechanisms underlying the occurrence and development of depression (62). In fact, physical activity of any level can help prevent and treat depression in adults and adolescents, and the longer the intervention duration and the higher the activity intensity, the better the antidepressant effect (63, 64). Conversely, when LPA, MVPA, or SLP replaced ST, depressive symptoms showed significant improvement. However, the substitution effects of different activity behaviors were asymmetrical. For instance, LPA replacing ST resulted in a more moderate improvement in depression scores, whereas ST replacing LPA led to a more significant increase in depression scores, consistent with previous research findings (65). Additionally, when LPA replaced MVPA, depression scores showed a positive upward trend, suggesting that LPA may not be sufficient to significantly improve depressive symptoms. When NSST replaced PA or SLP, depressive symptoms were also exacerbated, possibly due to the health risks associated with NSST, such as cardiovascular and metabolic issues, loneliness, etc. (66–68).

When analyzing the effect of sleep substitution on depression, it must be interpreted in conjunction with the actual sleep status of adolescents. The paradoxical finding that vigorous physical activity with sleep replacement was associated with an increase in depression scores may be due to the fact that sleep deprivation was common among the participants: only 42.06% of junior high school students and 29.70% of senior high school students achieved the recommended 9 h of sleep. In adolescents who are already in sleep debt, replacing exercise with sleep increases rest but may lose the antidepressant effects of exercise, resulting in a negative net effect. In contrast, replacing screen time with sleep was associated with lower depression scores, suggesting a benefit for sleep-deprived adolescents, both to compensate for sleep deficits and to reduce the interference of nocturnal blue light exposure with circadian rhythms. These findings suggest that sleep interventions should be tailored to individuals, with priority given to those with severe sleep deficit and emphasis on maintaining adequate physical activity for those with adequate sleep.

### 5.6 Methodological contributions and implications

This study makes an important methodological contribution to the analysis of the relationship between 24-h activity behaviors and depressive symptoms among adolescents. One of the core innovations of this study is the adoption of the CISM, which incorporates screen time as an independent and separate behavioral component into the analysis of 24-h activity composition, rather than simply classifying it into the broad category of “sedentary behavior”.

Most previous relevant studies typically treated sedentary behavior as a single homogeneous construct, combining screen time and non-screen sedentary time into a single indicator for analysis (69, 70). Such an approach masks the fundamental differences between screen-based sedentary behavior and non-screen sedentary behavior in terms of psychological mechanisms and health impacts, making it difficult to accurately reveal the unique effect of screen use itself on mental health. In contrast, the CISM employed in this study can account for the constrained nature of 24-h time-use data and simultaneously estimate the temporal substitution effects among five mutually exclusive behaviors: MVPA, LPA, ST, NSST, and SLP. By isolating ST for independent analysis, this study more precisely identifies how increases or decreases in screen time, and its displacement of other behaviors, independently influence depressive symptoms in adolescents without interference from other sedentary or active behaviors.

In summary, by using the CISM to analyze screen time as an independent behavioral component, this study represents a methodological improvement in research on 24-h activity patterns and mental health. This approach enhances the explanatory power of time allocation effects and provides a methodological reference for subsequent more precise and targeted intervention studies. By disentangling screen time from the broad category of sedentary behavior, this study offers more detailed and robust evidence for understanding the relationship between screen use and depression among adolescents, demonstrating the originality and innovative value of this research in both methodology and findings.

## 6 Limitations

This study provides new insights into adolescent depression interventions and clarifies the potential impact of the flexible and holistic allocation of activity time on mental health, yet it has several inherent limitations that may affect the interpretation and generalization of the results: first, there are measurement errors arising from self-reported questionnaires and methodological limitations of assessment tools, as all core research indicators were collected via self-administered questionnaires which are susceptible to recall bias that may introduce systematic errors into the data and reduce the reliability of the correlation analysis between activity behaviors and depression scores, and the Chinese version of the IPAQ-SF used sets a recording threshold of at least 10 min per session, leading to a severe underestimation of fragmented low-intensity physical activity (LPA) among adolescents while moderate-to-vigorous physical activity (MVPA) is mostly continuous and thus minimally affected by this measurement bias, which may result in deviations between the estimated duration of LPA and the analysis of its relevant correlations and the actual situation; second, the cross-sectional study design limits the inference of causal relationships, as the study only reveals the correlations between various behavioral factors and depressive symptoms at a single time point, failing to confirm the direction of causal relationships and rule out the possibility of reverse causality, and it also cannot capture the dynamic changes in activity behaviors and depressive symptoms, making it difficult to explore the long-term causal effects of behavioral changes on depression; third, the research sample has limited representativeness that restricts the generalization of results, for study participants were only recruited from urban areas of Hefei City, and there are significant differences in economy, culture, education and adolescent lifestyles between urban and rural areas as well as among the eastern, central and western regions of China, with notable differences also existing in the characteristics of adolescents from different cultural backgrounds overseas, meaning the conclusions cannot be directly generalized to other groups and their applicability needs to be verified by subsequent research; fourth, residual confounding factors may affect the accuracy of result estimation, because although a series of demographic and socioeconomic confounding variables such as gender, grade and household income were controlled for in the statistical analysis, unmeasured psychological and social factors including family history of mental illness, peer relationships, school pressure and parent-child attachment may still interfere with the correlation between activity behaviors and depression, leading to residual confounding bias and thus the overestimation or underestimation of relevant correlations.

## 7 Conclusion

This study applied the compositional isotemporal substitution model (CISM) to explore the relationship between adolescent depression scores and screen time (ST), physical activity (PA) and sleep (SLP). The results indicated that ST, PA and SLP all exert significant effects on adolescent depression, and the time reallocation among these behaviors has a profound impact on depression scores. Specifically, ST is a risk factor for adolescent depression, while low-intensity physical activity (LPA), moderate-to-vigorous physical activity (MVPA) and SLP all act as protective factors. Notably, replacing ST with MVPA is the most effective time reallocation strategy for alleviating adolescent depressive symptoms. Conversely, substituting ST for MVPA, LPA or SLP leads to a significant increase in depression scores, with this adverse effect amplifying as the substitution duration lengthens. Based on these findings, reducing ST and increasing MVPA should be prioritized in adolescent mental health interventions. We can foster a healthy behavioral pattern of less screen time, sufficient sleep and more physical activity by having schools add physical activities and formulate holistic 24-h activity time plans, providing personalized guidance for high-risk groups, and strengthening health education.

## Data Availability

The original contributions presented in the study are included in the article/supplementary material, further inquiries can be directed to the corresponding authors.
